# Effect of the 5-HT_4_ receptor agonist tegaserod on the expression of GRK2 and GRK6 in the rat gastrointestinal tract

**DOI:** 10.1186/s13104-018-3495-2

**Published:** 2018-06-08

**Authors:** Teshome Nedi, Paul J. White, Ian M. Coupar, Helen R. Irving

**Affiliations:** 1 Monash Institute of Pharmaceutical Sciences, Monash University (Parkville Campus), 381 Royal Parade, Parkville, VIC 3052 Australia; 2 School of Pharmacy, College of Health Sciences, Addis Ababa University, PO Box 1176, Addis Ababa, Ethiopia; 3 La Trobe Institute for Molecular Science, La Trobe University, PO Box 199, Bendigo, VIC 3552 Australia

**Keywords:** Colon, Oesophagus, 5-HT_4_ receptors, G protein coupled receptor kinases, Receptor desensitization

## Abstract

**Objective:**

Tegaserod is a 5-hydroxytryptamine type 4 (5-HT_4_) receptor agonist, formerly used in treating constipation predominant irritable bowel syndrome, which desensitizes 5-HT_4_ receptors in rat oesophagus and colon in vitro. Desensitization of 5-HT_4_ receptors is regulated by G-protein coupled receptor kinases. This study was designed to assess the effect of 5-HT_4_ receptor activation on the expression of GRK2 and GRK6 in the rat oesophagus and distal colon by acute administration of tegaserod.

**Results:**

Rats were treated with a single dose of tegaserod (5 mg/kg) and tissue samples of the oesophagus and distal colon were prepared and level of GRK2 and GRK6 protein expression was determined using western blotting. The immunodensity of GRK2 and GRK6 was normalized against the loading control β-actin and compared with control animals. Acute administration of tegaserod for 1, 2, 3, 4, 6, and 8 h did not change significantly the immunodensity of GRK2 or GRK6 in the oesophagus or GRK2 in the distal colon when compared with control animals. This may indicate that the basal level of GRK2 and GRK6 expression is sufficient to regulate the desensitization of 5-HT_4_ receptors in acute drug treatment.

## Introduction

5-Hydroxytryptamine type 4 (5-HT_4_) receptor agonists have prokinetic effects in the gastrointestinal tract stimulating motility and secretion through enhanced acetylcholine release from excitatory motor neurons and interneurons [1]. Tegaserod is a 5-HT_4_ receptor agonist with clinical efficacy in patients with constipation-predominant irritable bowel syndrome and chronic constipation [2]. Tegaserod was used for the treatment of constipation-predominant irritable bowel syndrome in females and chronic constipation for both males and females until withdrawn in 2007 as it was associated with rare adverse cardiovascular effects [2, 3]. Tegaserod increases gastric emptying and accelerates small intestine and colonic transit in healthy human subjects [4] but increases colonic transit without altering gastric emptying in patients with constipation-predominant irritable bowel syndrome [5]. Tegaserod has a low therapeutic gain of 5–12% above placebo and is poorly absorbed with about two-thirds of oral doses being eliminated in the faeces [6]. It facilitates the peristaltic reflex in mouse, rat, guinea-pig and human intestine and also attenuates sensory neurotransmission in human rectum [7–12]. Tegaserod increased the amplitude of evoked excitatory postsynaptic currents in cultured myenteric neurons from mice, which desensitized rapidly, making it difficult to obtain responses to higher concentrations [13]. In addition, tegaserod desensitizes 5-HT_4_ receptors in rat oesophagus and colon in vitro [14, 15].

G protein coupled receptor (GPCR) kinase (GRK) regulated homologous desensitization has been reported for 5-HT_4_ receptors in mouse colliculus neurons and rat oesophagus and colon [14–17]. Cell culture studies using co-expression of GRKs with different 5-HT_4_ receptor splice variants showed the involvement of GRK2 and GRK5 in desensitization [18–20]. In contrast, desensitization of 5-HT_4_ receptors has been associated with GRK6 in the oesophagus and both GRK2 and GRK6 in the distal colon of the rat [15].

Changes in the level of GRK expression elicited by in vivo stimulation or blockade of GPCRs by agonist and antagonist is complex and not predictable [21, 22]. For example, chronic treatment with desipramine (a noradrenaline reuptake blocker) and acute treatment with fluoxetine (a 5-HT reuptake blocker) did not change significantly the immunodensity of GRK2/3 [23]. While chronic treatment with both opioid agonists and antagonists increases the expression of GRK2 and GRK6 in the brain [24, 25]. The molecular mechanisms associated with the regulation of 5-HT_4_ receptors in vivo by specific GRKs after treatment with 5-HT_4_ receptor agonist has not been studied. Therefore, the aim of this study was to assess the effect of 5-HT_4_ receptor activation on the expression level of GRK2 and GRK6 in the rat oesophagus and distal colon by acute administration of tegaserod.

## Main text

### Methods

Adult male Sprague–Dawley rats (Monash Animal Services) weighing 200–280 g were randomly divided into six treatment groups and one control group (n = 6; total number = 42). Rats were housed at the Faculty of Pharmacy and Pharmaceutical Sciences, Monash University’s animal facility in a 12 h light–dark cycle with food and water ad libitum. Rats were treated with a single intraperitoneal injection of tegaserod maleate (Sequoia Research Products Ltd, Berkshire, UK) (5 mg/kg) dissolved in vehicle (10% sulfobutyl ether-beta cyclodextrin) or just vehicle (controls). Rats were killed 1 h after vehicle or 1, 2, 3, 4, 6 and 8 h after drug administration by carbon dioxide asphyxiation. Animals were quickly dissected and the lower third of the oesophagus proximal to the diaphragm, and the distal colon were excised. The outer muscularis externa of the oesophagus was separated from the inner tunica muscularis mucosae. Tissues were washed three times with ice-cold phosphate buffered saline (PBS) and homogenized in 1:10 (w/v) of homogenization buffer [50 mM Tris–HCl, pH 7.5, 150 mM NaCl, 1% Nonidet P40, 0.5% sodium deoxycholate, complete protease inhibitor cocktail tablet (Roche, Sydney, Australia)] for 1 min before centrifugation at 12,000×*g* for 10 min. Supernatant protein concentration was determined by Quant-It protein assay (Invitrogen) and homogenates were stored at − 80 °C until used.

Total protein (50 µg) was separated on 12% polyacrylamide gels by SDS-PAGE and transferred to nitrocellulose membranes (Amersham Bioscience). Each gel was run with a sample from control to all test groups. Nonspecific binding to the membrane was blocked with Odyssey blocking buffer (LI-COR Biosciences) for 1 h. Membranes were probed with combination of two primary antibodies: goat anti-β-actin and either mouse anti-GRK2 or rabbit anti-GRK6 (Table 1) overnight at 4 °C and washed four times with PBS and 0.1% Tween 20 PBS (PBST). Membranes were incubated with fluorophore-conjugated secondary antibodies (Table 1) for 1 h at room temperature and washed four times for five min using PBST. An Odyssey Infrared Imaging System (LI-COR Biosciences) was used to examine the immunoblots. The integrated optical density of the immunoreactivity was assessed using the Odyssey Infrared Imaging System software (LI-COR Biosciences). The integral optical density of GRK2 and GRK6 from each lane was normalised against β-actin for that lane and expressed as percentage of control values.

**Table 1:** Details of primary and secondary antibodies

Antigen	Host species	Dilution	Sources	References
β-Actin	Goat	1:500	Abcam	
GRK2	Mouse	1:500	Santa Cruz; sc-13143	[15, 36–38]
GRK6	Rabbit	1:300	Santa Cruz; sc-566	[15, 21, 39, 40]
Anti-mouse IRDye 800CW	Donkey	1:10,000	LI-COR Biosciences; 925-32212	
Anti-goat IRDye 680RD	Donkey	1:10,000	LI-COR Biosciences; 925-68074	
Anti-rabbit IRDye 800CW	Donkey	1:10,000	LI-COR Biosciences; 925-32213	

Data for each treatment group was expressed as mean ± standard error of mean (SEM) of GRK2 and GRK6 levels expressed as percentage of control values. Statistical analyses were performed using GraphPad Prism 5 (GraphPad Software, La Jolla, California USA). Values were compared between the different groups using one-way analysis of variance (ANOVA) followed with Dunnett’s multiple comparison post hoc test. A *P* value < 0.05 was considered to be statistically significant.

### Results

**Fig. 1:**
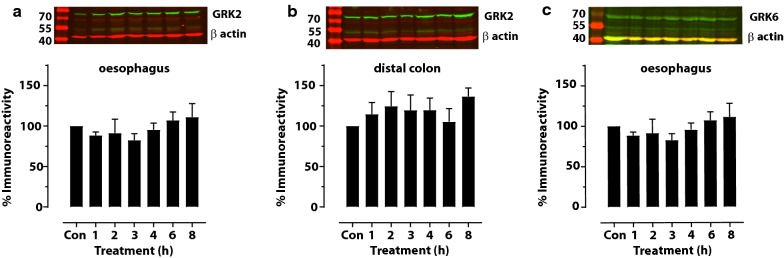
Time course study of the effect of tegaserod (5 mg/kg, i.p.) on the expression levels of GRK2 and GRK6 in the rat digestive tract. **a** GRK2 levels in the oesophagus; **b** GRK2 levels in the distal colon; and **c** GRK6 levels in the oesophagus. Upper panels: representative immunoblots illustrating the effects of tegaserod on the expression level of GRK2 or GRK6 at 1, 2, 3, 4, 6 and 8 h after treatment. Lower panels: the mean densitometric values of GRK2 or GRK6 levels relative to β-actin and expressed as percentages of values in untreated control rats (± SEM, n = 6 per group). No significant difference from the control occurred in any of the treatments (P > 0.05; one-way ANOVA). *Con* control, *h* hours following treatment

Immunoblot analysis of the tissue homogenate of the oesophagus and distal colon revealed immunoreactive protein of around 80 kDa for GRK2 or alternatively 66 kDa for GRK6 under the 800 nm excitation wavelength channel and around 42 kDa for β-actin under the 700 nm excitation wavelength channel on the same membrane (Fig. 1). This allowed the analysis of the immunodensity of GRK2 and β-actin or GRK6 and β-actin on the same membrane. The immunodensity of GRK2 relative to β-actin did not change significantly in either the oesophagus (P = 0.52, one-way ANOVA) or distal colon (P = 0.66, one-way ANOVA) following acute treatment with tegaserod (Fig. 1a, b). Similarly, the immunodensity of GRK6 relative to β-actin in the oesophagus (Fig. 1c) not change significantly (P = 0.92, one-way ANOVA) following acute treatment with tegaserod.

### Discussion

This study was designed to investigate the effect of acute activation of 5-HT_4_ receptors using the 5-HT_4_ receptor agonist tegaserod on the expression level of GRK2 and GRK6 in rat oesophagus and distal colon. Prior studies indicate that in the oesophagus GRK6 is associated with the desensitization of 5-HT_4_ receptors whilst GRK2 and GRK6 are associated with desensitization of 5-HT_4_ receptors in the distal colon of the rat [15]. GRK-mediated GPCR desensitization has a physiological significance to protect cells from over-stimulation in the persistent presence of agonists and to keep the signals under regulation. Up-regulation of GRKs facilitates GPCR desensitization whilst down-regulation of GRKs impedes GPCR desensitization in vitro and in vivo [26]. Intravenous administration of tegaserod (0.001–1 mg/kg) has been reported to evoke a dose-dependent increase in relaxation of the longitudinal muscle of the rat oesophagus. The response had a rapid onset (commencing less than 1 min after dosing) and reached a maximum typically within 2 min [27–29]. In addition, subcutaneous administration of tegaserod (0.03, 0.3 and 3 mg/kg) has been reported to produce a dose-dependent colonic prokinetic effect in guinea-pigs [30]. Moreover, intraperitoneal administration of tegaserod (0.1, 1, 10 mg/kg) has been reported to produce a dose-dependent reduction in the number of abdominal contractions induced by colonic distensions in rats [8]. Based on the above studies a single dose of tegaserod (5 mg/kg i.p.) was used for time course study of the effect of 5-HT_4_ receptor activation on the level of expression of GRK2 and GRK6 in the rat oesophagus and distal colon.

Similar to the finding of the present in vivo study, acute treatment with the 5-HT reuptake blocker fluoxetine that increases the synaptic concentration of 5-HT, was not associated with changes in the immunodensity of GRK2/3 in rat frontal cortex membrane and cytosolic fractions [23]. In contrast, acute treatment with tricyclics antidepressant desipramine increased in a dose and time-dependent manner the content of GRK2/3 in the membrane whilst chronic treatment did not alter the immunodensity of GRK2/3 [23].

Tegaserod activates 5-HT_4_ receptors on intrinsic primary afferent neurons and mimics the action of endogenous serotonin released from enterochromaffin cells. It stimulates the intrinsic primary afferent neurones that release transmitters such as calcitonin gene-related peptide, which activate cholinergic interneurons. The cholinergic interneurons activate the peristaltic reflex [9]. Tegaserod has been shown to desensitize the peristaltic reflex induced by mucosal stimulation in a time and concentration dependent manner in rat colon [14]. In addition, in rat oesophagus, tegaserod desensitized 5-HT induced-relaxation of the tunica muscularis mucosae in a time-dependent manner [15]. The lack of change in the expression level of GRK2 in the oesophagus (Fig. 1a) is in line with the immunohistochemistry study showing that there was no expression of GRK2 on the smooth muscle of the muscularis mucosae where the 5-HT_4_ receptors are concentrated in the oesophagus [15, 31, 32]. The interesting finding is the lack of change in the expression level of GRK6 in the oesophagus and GRK2 in distal colon tissues (Fig. 1b, c) where they were found to be co-expressed and co-immunoprecipitated with 5-HT_4_ receptors [15]. This may be due to the expression of sufficient levels of GRK2 and GRK6 at the basal level to regulate the desensitization of 5-HT_4_ receptor. Alternatively, it may require chronic administration of tegaserod to obtain changes in the expression level of GRK2 and GRK6. The desensitization of GPCRs requires translocation of GRK2 from cytosol to membrane upon activation by agonist [33]. Analysis of change in the immunoreactivity of GRK2 in the membrane fraction warrant further investigation.

In the distal colon, 5-HT_4_ receptor immunoreactive cells were found in longitudinal muscle, myenteric plexuses, circular muscle, submucosal plexuses and muscularis mucosae. GRK6 was expressed in the longitudinal muscle, circular muscle, and muscularis mucosae and co-immunoprecipitated with 5-HT_4_ receptors [15]. Due to the interference of the background it was not possible to quantify the immunodensity of GRK6. In addition, the 5-HT_4_ receptors are located presynaptically at neuronal synapses within the myenteric plexus [1] and GRK5 is exclusively localised on the nerve endings of both myenteric and submucosal plexuses [15]. It is therefore worthwhile to investigate the effect of tegaserod on the expression of GRK5 and GRK6 at mRNA level in the distal colon.

Based on desensitization studies of β_2_-adrenoceptors and M_3_ muscarinic receptors, it is often generalized that partial agonists induce less desensitization of GPCRs than full agonists. They could stabilize receptor conformations that differ in their capacity to interact or serve as substrates for GRKs and arrestins to generate downstream recognition barcodes [34, 35]. Partial agonists augment submaximal endogenous stimulation and prevent an exaggerated response to an endogenous agonist. As a result partial agonists have a lower tendency to induce receptor desensitization and/or receptor down-regulation [34, 35]. However, the capacity of agonists to induce desensitization of 5-HT_4_ receptors depends more on the activation potency of the drug than its efficacy [16]. The lower potency of tegaserod may contribute to the lack of its effect on the expression level of GRKs in rat oesophagus and distal colon. Alternatively, tegaserod has similar binding affinities to both 5-HT_4_ and 5-HT_2B_ receptors [30] and acts as an antagonist at the 5-HT_2B_ receptor [29, 30]. Thus, the 5-HT_2B_ receptor antagonist effect of tegaserod may counteract its effect on the expression level of GRKs.

## Limitations

Limitations of this study include the use of only one concentration of tegaserod. However, the concentration used reduces the number of abdominal contractions induced by colonic distensions in rats measured over 90 min [8]. Taken together with prior observations that desensitization is only detectable after 20 min in rat oesophagus [15], our sampling times of 1–8 h should detect changes in GRK expression. Another limitation is the use of a control at only a single time point, which occurred in part to restrict animal usage. A further limitation is the lack of positive control for changes in GRK2 and 6 expression although our prior study [15] indicated that the antisera used detected changes in the expression level of these proteins. So far, no studies have investigated the effect of 5-HT_4_ receptor activation on the expression level of GRKs using agonists with different potency, selectivity and efficacy. Such studies will help understand whether the expression of GRKs is altered based on agonist potency, selectivity or efficacy in physiological systems.

## Abbreviations

5-HT_4_: 5-hydroxytryptamine type 4
ANOVA: one-way analysis of variance
GPCR: G-protein coupled receptor
GRK: GPCR kinase
PBS: phosphate buffered saline
PBST: phosphate buffered saline Tween
SEM: standard error of mean
